# A few-layer graphene/chlorin e6 hybrid nanomaterial and its application in photodynamic therapy against *Candida albicans*

**DOI:** 10.3762/bjnano.11.90

**Published:** 2020-07-17

**Authors:** Selene Acosta, Carlos Moreno-Aguilar, Dania Hernández-Sánchez, Beatriz Morales-Cruzado, Erick Sarmiento-Gomez, Carla Bittencourt, Luis Octavio Sánchez-Vargas, Mildred Quintana

**Affiliations:** 1 Centro de Investigación en Ciencias de la Salud y Biomedicina, Universidad Autónoma de San Luis Potosí, México; 2 Chimie des Interactions Plasma – Surface (ChIPS), Research Institute for Materials Science and Engineering, Université de Mons, Belgium; 3 Instituto de Física, Universidad Autónoma de San Luis Potosí, San Luis Potosí, SLP, Mexico; 4 GRAPHENEMEX S.A. de C.V., Ciudad de México, México; 5 CONACYT – Universidad Autónoma de San Luis Potosí, San Luis Potosí, SLP, México; 6 Departamento de Ingeniería Física, División de Ciencias e Ingenierías, Universidad de Guanajuato, León, Guanajuato, México; 7 Laboratorio de Bioquímica, Patología y Microbiología, Facultad de Estomatología, Universidad Autónoma de San Luis Potosí, México; 8 Facultad de Ciencias, Universidad Autónoma de San Luis Potosí, México

**Keywords:** chlorin e6, few-layer graphene (FLG), hybrid nanomaterial, photodynamic therapy (PDT), photosensitizer

## Abstract

The global emergence of multidrug resistance of fungal infections and the decline in the discovery of new antibiotics are increasingly prevalent causes of hospital-acquired infections, among other major challenges in the global health care sector. There is an urgent need to develop noninvasive, nontoxic, and new antinosocomial approaches that work more effectively and faster than current antibiotics. In this work, we report on a biocompatible hybrid nanomaterial composed of few-layer graphene and chlorin e6 (FLG-Ce6) for the photodynamic treatment (PDT) of *Candida albicans*. We show that the FLG-Ce6 hybrid nanomaterial displays enhanced reactive oxygen species (ROS) generation compared with Ce6. The enhancement is up to 5-fold when irradiated for 15 min at 632 nm with a red light-emitting diode (LED). The viability of *C. albicans* in the presence of FLG-Ce6 was measured 48 h after photoactivation. An antifungal effect was observed only when the culture/FLG-Ce6 hybrid was exposed to the light source. *C. albicans* is rendered completely unviable after exposure to ROS generated by the excited FLG-Ce6 hybrid nanomaterial. An increased PDT effect was observed with the FLG-Ce6 hybrid nanomaterial by a significant reduction in the viability of *C. albicans*, by up to 95%. This is a marked improvement compared to Ce6 without FLG, which reduces the viability of *C. albicans* to only 10%. The antifungal action of the hybrid nanomaterial can be activated by a synergistic mechanism of energy transfer of the absorbed light from Ce6 to FLG. The novel FLG-Ce6 hybrid nanomaterial in combination with the red LED light irradiation can be used in the development of a wide range of antinosocomial devices and coatings.

## Introduction

The frequency of fungal infections has notably increased in the last decades; for instance, *Candida albicans* is now reported as the fourth cause of nosocomial septicemia in the United States [1]. Among the reasons behind the increase of incidences of fungal infections are the indiscriminate use of antineoplastic and immunosuppressive drugs, the unnecessary use of broad-spectrum antibiotics, the growing application of prosthetic devices, and the increased number of invasive surgeries [2–3]. The prevailing drugs used to fight fungal infections usually require long treatments and very often present side effects [2]. For these reasons, improved antifungal therapies must be developed to treat fungal infections [4].

An alternative approach, used as medical technology to treat diseases like cancer and fungal infections, employs visible light to activate photosensitive molecules, known as photodynamic therapy (PDT) [4]. PDT was discovered in 1900 when *Paramecia* microorganisms were exposed to a photosensitive molecule in conjugation with sunlight, which was found to eliminate the fungal activity of *Paramecia* [5]. PDT consists of the interaction of visible-light photons with a photosensitizer located inside the cell or in close proximity to it. In this interaction, the photosensitizer produces highly reactive oxygen species (ROS) by reacting in the excited state with molecular oxygen present in the environment. ROS refer to molecules like singlet oxygen, superoxide anion, and radicals, which are responsible for producing oxidative stress in cells followed by cell death [4]. Photosensitizer molecules must be nontoxic before irradiated with light, must produce high amounts of ROS when irradiated with visible light, and also should have a high absorption coefficient at a wavelength that penetrates cellular tissue [4]. In the visible electromagnetic spectrum, red light has the largest depth penetration into biological tissue [6–7]. Among different photosensitizers, porphyrins are heterocyclic molecules derived from four pyrrole-like subunits interconnected by methine groups [8]. Porphyrins possess all the characteristics of a good photosensitizer since they are planar or semiplanar molecules with π-electrons in a closed ring, resulting in a large conjugated system that strongly absorbs light in the visible spectra. An example of this is the porphyrin chlorin e6 (Ce6), which has been widely used as a photosensitizer in PDT [9–13]. One of the main drawbacks in the use of Ce6, and in general for any other organic photosensitizer materials, is the quenching after photoexcitation which results in the decay of ROS production due to molecular degradation. There have been several attempts to enhance the PDT properties of organic molecules like Ce6 by the preparation of composites using nanoparticles [14–16]. Such hybrid nanomaterials take advantage of both the good photosensitizer properties of Ce6 and of the physical and chemical characteristics of the nanoparticle. Consequently, great improvements are expected when the synergistic characteristics from the hybrid nanomaterial constituents complement each other, turning the system into a more useful tool for a diverse number of biological applications, such as biosensors, protein detection, bioimaging and drug delivery [17–18].

In recent years, graphene nanoparticles have been used in many different applications ranging from enhanced spectroscopy techniques, coatings, polymeric composites, sensors, drug delivery systems and others, due to their excellent physical and chemical properties (e.g., high surface area, excellent thermal and electric conductivity, high mechanical strength)[19–21]. Examples of graphene nanomaterials include single-layer graphene, few-layer graphene (FLG), graphene oxide (GO), and the reduced form of GO (rGO) [22]. GO and rGO have been conjugated to several photosensitizers to enhance their performance in PDT [15,17,23–25]. However, for enhancing the characteristics of a Ps, the properties of graphene, such as electrical conductivity and chemical stability are very important, and these properties are significantly hindered in GO and rGO [26–29]. Thus, the conjugation of pristine graphene with photosensitizer molecules might result in a more efficient and stable material for PDT.

In this work, FLG combined with Ce6 was used as a photosensitizer in PDT as an antifungal treatment against *C. albicans*. *Candida albicans* is the most virulent *Candida* specie with a high economic and medical relevance due to high health care cost, in addition to high morbidity and mortality rates, especially in immunocompromised patients [30]. As a result, *C. albicans* is commonly used to test different materials as candidates for photosensitizers in PDT [31–32]. An FLG and Ce6 hybrid nanomaterial (FLG-Ce6) was used as the photosensitizer in combination with a red light-emitting diode (LED) array as the photoactivation light source. The conjugated system of graphene π-electrons improves the performance of Ce6 through the donation of electrons that delay its photobleaching. In this way the production of reactive oxygen species is optimized and a better effect against *C. albicans* is achieved with a low concentration of photosensitizer and a short exposure time to the red LED light source.

## Results and Discussion

The liquid phase exfoliation of graphite was first carried out in toxic, non-biocompatible solvents due to the match in the surface energy of graphene and the solvents [33]. However, the interest in using graphene for biological applications has led to the development of new synthetic techniques, such as the exfoliation of graphene assisted by molecules that display amphiphilic properties [34–35]. These molecules are intercalated between the graphene sheets, conferring stability and solubility in media in which graphene by itself is not soluble.

In 2016, Hernández et al. [36] reported the synthesis of FLG by the exfoliation of graphite in water and phosphate buffer saline (PBS) using Ce6 as the stabilizing molecule. The π–π stacking interactions between FLG and Ce6 allows the stabilization of FLG in biocompatible media. Following this methodology, a FLG-Ce6 hybrid nanomaterial was prepared by the exfoliation of graphite in sterile deionized water using Ce6 as the stabilizing molecule. To do this, graphite was sterilized by exposure to ultraviolet light for 45 min, and a methanol solution of Ce6 was filtered using a 0.2 μm pore size filter to ensure the sterility of the sample. The concentration of Ce6 in the FLG-Ce6 hybrid was calculated using UV–vis absorption spectroscopy. Ce6 presents an intense absorption band at 407 nm, corresponding to the Soret band. A Ce6 calibration curve at this wavelength is then produced. Then, in order to obtain a correct approximation of the real concentration of Ce6 in FLG-Ce6, the absorption of FLG at 407 nm, under the same experimental conditions, is subtracted from the absorption obtained from the solution of FLG-Ce6 and finally matched with the calibration curve of Ce6.

**Figure 1:**
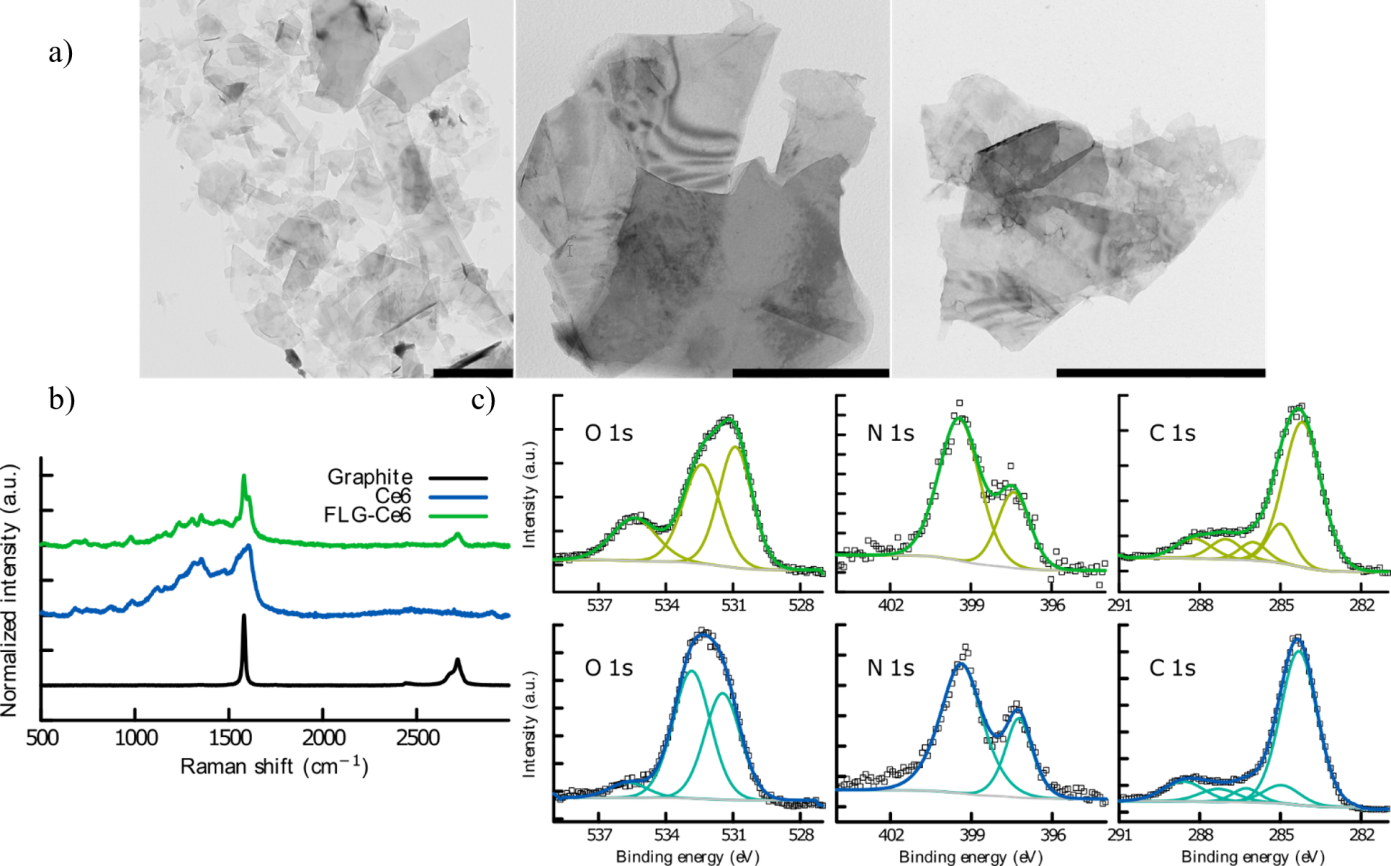
Characterization of the FLG-Ce6 hybrid nanomaterial. a) TEM images of FLG-Ce6; scale bar is 1 µm. b) Raman spectrum of FLG-Ce6, Ce6 and graphite. c) XPS spectra. Top row (green) shows the FLG-Ce6 chemical analysis and bottom row (blue) shows the respective analysis for Ce6.

Figure 1a shows transmission electron microscopy (TEM) images of FLG-Ce6. Graphene sheets exfoliated from graphite are evidence of the interaction between FLG and Ce6 in pure water. Ce6 stabilizes graphene sheets, avoiding aggregation due to its high attraction through π–π stacking interactions, and the oxygenated moieties on Ce6 allow the stabilization of the hybrid nanomaterial in water. The Raman spectra of the hybrid nanomaterial, Ce6 and graphite are shown in Figure 1b for comparison. The highest peak, corresponding to the G band (≈1580 cm^−1^), was used to normalize the signals in each case. The 2D band (≈2715 cm^−1^) gives information about π–π stacking which only occurs in graphite. The intensity of the 2D band is reduced in the FLG-Ce6 hybrid nanomaterial due to the interaction between FLG and Ce6 π-electrons. Finally, the D band (≈1350 cm^−1^) is not present in the Raman spectrum of pristine graphite as it gives information about the in-plane defects of the graphene lattice. In the Raman spectrum of the FLG-Ce6 hybrid nanomaterial, the D band is overshadowed by the Raman signals of Ce6.

Figure 1c shows the X-ray photoelectron spectroscopy (XPS) spectra of the hybrid nanomaterial and Ce6. The O 1s core level spectrum of the hybrid nanomaterial FLG-Ce6 is mainly composed of two peaks at 530.9 and 532.4 eV corresponding to HO–C and C=O, respectively. There is a peak at 535.4 eV corresponding to the chemisorbed oxygen on the hybrid nanomaterial, which is almost absent in the Ce6 sample. The N 1s core level spectrum is the same in the hybrid nanomaterial as in the Ce6 sample because the nitrogen contribution comes only from Ce6 in both cases. The spectrum analysis allows the two types of chemical bonding of nitrogen present in the Ce6 structure to be distinguished. The C 1s core level spectrum in the hybrid nanomaterial is mainly composed of a peak at 284.2 eV corresponding to C=C bonding. Additionally, components of carbon bonded to oxygen and nitrogen are present in the analysis, as expected.

**Figure 2:**
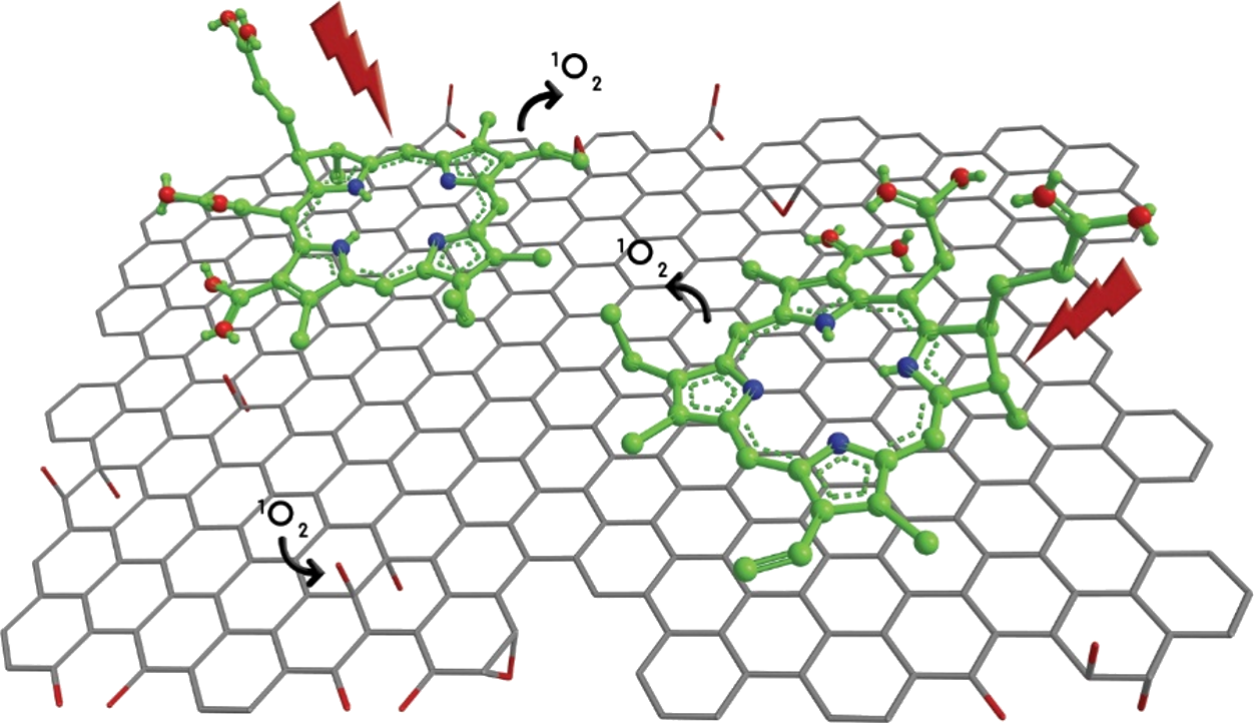
Schematic representation of the FLG-Ce6 hybrid nanomaterial. Ce6 molecules (green) stabilize a graphene sheet in water. FLG acts as an electron donor for the Ce6 molecules, enhancing its capability as a photosensitizer. The interaction of Ce6 molecules with the graphene sheet trough π–π stacking interactions prevents the quick photobleaching by shielding Ce6 from the interaction with the generated ROS.

Figure 2 shows a schematic representation of the FLG-Ce6 hybrid nanomaterial. The green dotted lines indicate the in-plane π–π stacking interactions between Ce6 and FLG. Red spheres represent the out-of-plane moieties responsible for the stabilization of the hybrid nanomaterial in water by the oxygen atoms.

**Figure 3:**
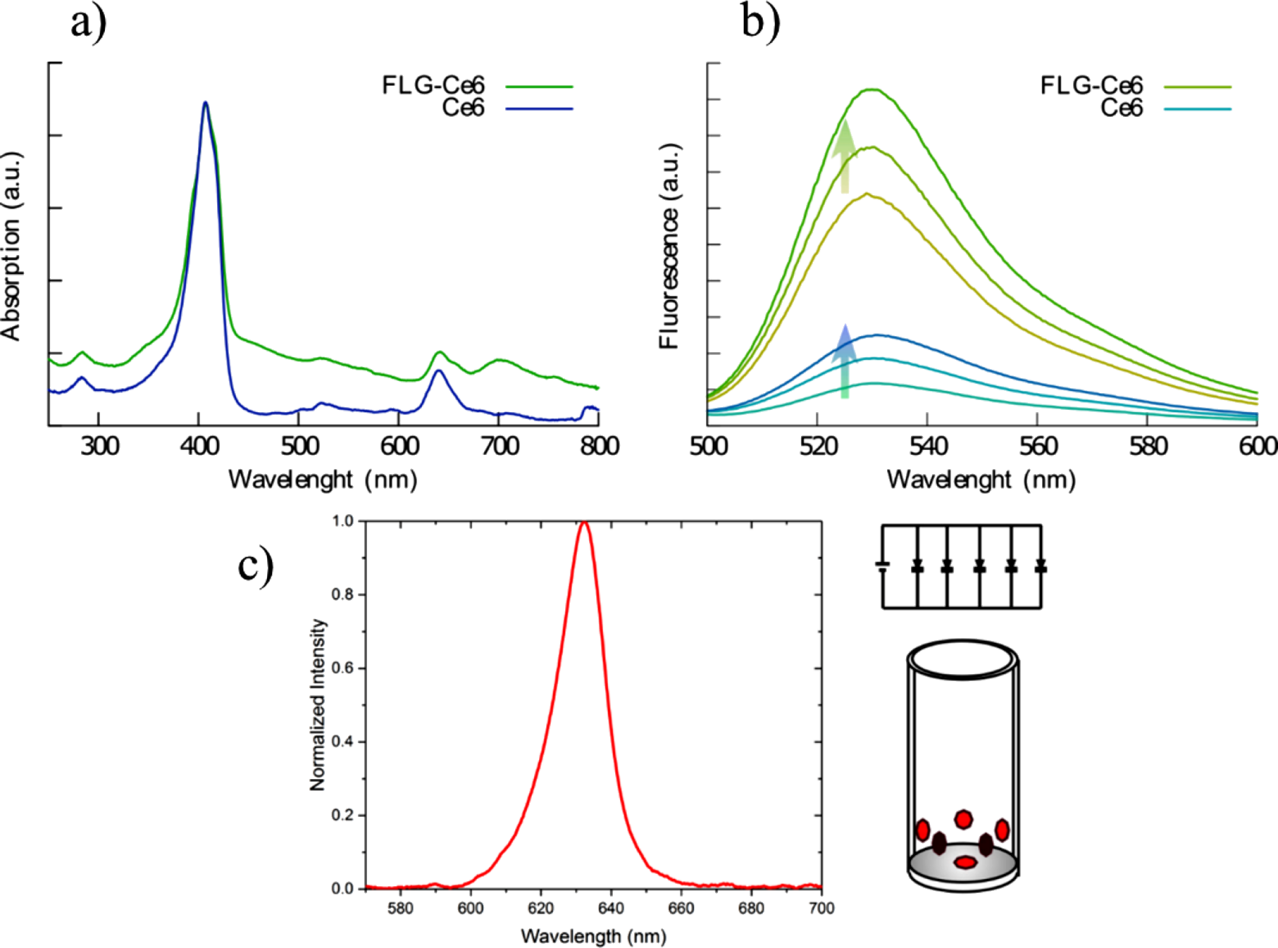
ROS production and PDT system characterization. a) UV–vis absorption spectra of pristine Ce6 (blue) and FLG-Ce6 (green). b) Singlet oxygen production assays. Singlet oxygen is indirectly observed through the fluorescence of the reporter molecule, singlet oxygen sensor green reagent; the higher fluorescence is related to higher singlet oxygen production. The ascending arrows denote the irradiation time (5, 10 and 15 min). c) Details of the LED excitation source. The LED emission spectrum is centered at 632 nm, as shown on the left, and a scheme of the 6 LEDs array is shown on the right.

Figure 3a shows the characterization results from UV–vis spectroscopy experiments on FLG-Ce6 and Ce6. The absorption spectrum of FLG-Ce6 shows absorption changes due to interactions between FLG and Ce6. A widening of the band at 400 nm and the appearance of a band at 700 nm were observed when compared with the Ce6 spectrum. The band at 700 nm appears to be due to π–π stacking interactions between FLG and Ce6. In a previous work [36], Ce6 was used for the stabilization of FLG and graphene oxide (GO). During the exfoliation of GO, the band at 700 nm was not observed since the conjugated system of π-electrons is highly compromised by the large amount of oxygen functionalities present in GO. The π–π stacking interactions between Ce6 and GO are negatively affected, resulting in the interaction by H-bond formation. Thus, FLG and Ce6 interact by means of π–π stacking, Ce6–Ce6 transition dipole, hydrogen bond formation, hydrophobic, and electron-donor interactions [36]. The observed changes in the UV–vis spectrum of FLG- Ce6 corroborates the TEM morphology observations.

The wider absorption peak in the FLG-Ce6 hybrid nanomaterial spectrum at 640 nm corresponds to the greater probability of absorbing a photon deeper into the tissue, since the penetration depth of electromagnetic radiation increases as the wavelength increases, reaching depths up to 3.5 mm at 1000 nm excitation [7]. Figure 3b shows the singlet oxygen production tests of FLG-Ce6 and pristine Ce6. The singlet oxygen production is indirectly observed through the fluorescence intensity of the singlet oxygen sensor green reagent (SOSG), which is a singlet oxygen reporter. Thus, FLG-Ce6 and pristine Ce6 samples were exposed to SOSG and photoactivated during 5, 10, and 15 min of visible light irradiation at 632 nm at an incident power of 150 mW. When analyzed using fluorescence spectroscopy, it was observed that ROS production is up to 5-fold higher in FLG-Ce6 than in pristine Ce6 and even greater at 15 min of photoactivation, with no evidence of photobleaching in any system. For this reason, tests with *C. albicans* were carried out at 15 min of exposure time.

The LED array used as the light source to photoactivate the FLG-Ce6 hybrid nanomaterial in the culture for the cellular viability tests was designed to have six LEDs that deliver approximately 150 mW of power at 632 nm through the center of the sample, as illustrated in Figure 3c.

**Figure 4:**
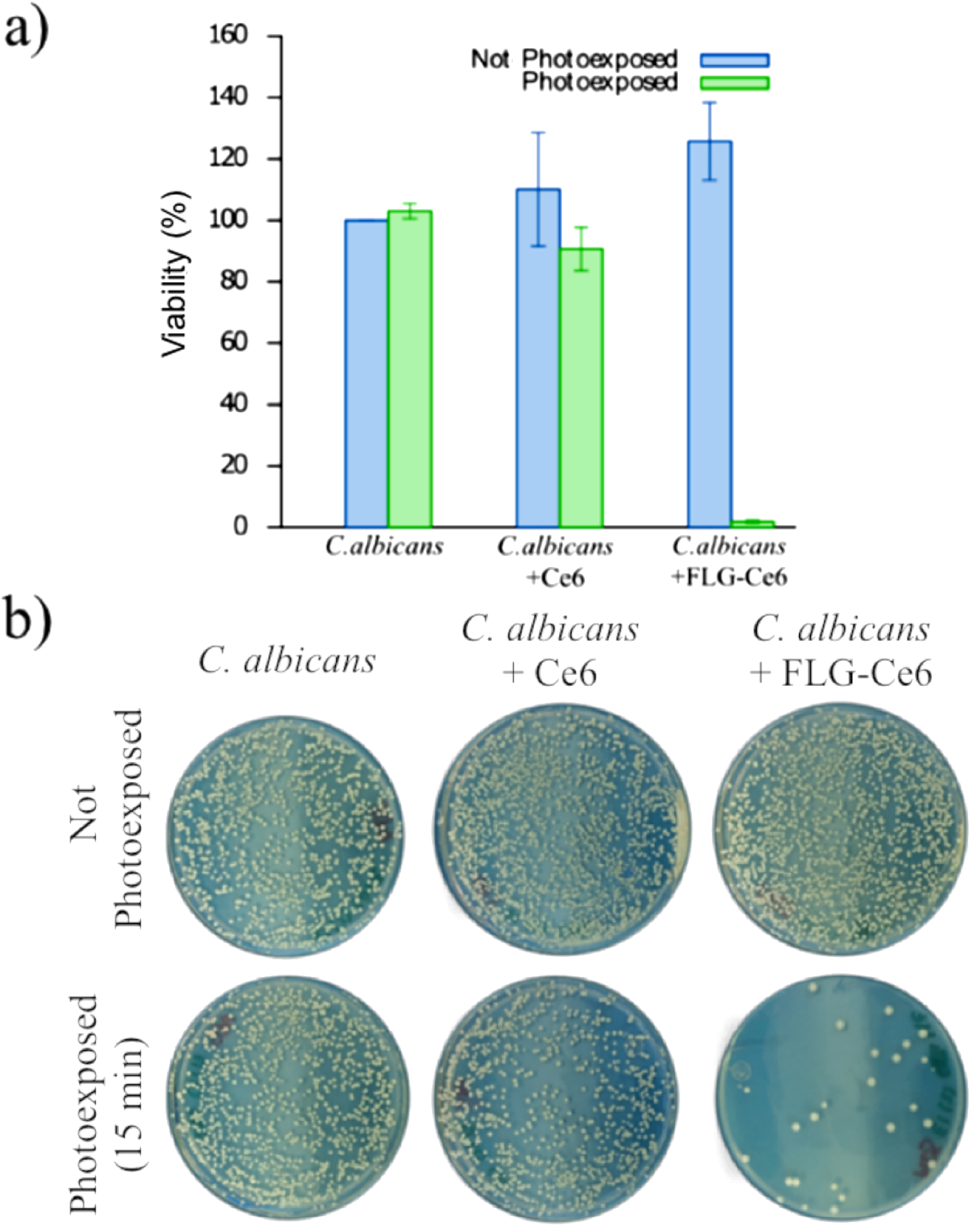
Cell viability assays. a) *C. albicans* viability 48 h after PDT. The cell cultures were exposed to the FLG-Ce6 hybrid nanomaterial and pristine Ce6 in different trials, and then the samples were photoactivated by illumination using the LED designed array for 15 min. b) An image of the colony forming units (CFUs) of *C. albicans* 48 h after PDT.

In Figure 4a, the viability of *C. albicans* is reported 48 h after being treated with PDT. The number of colony-forming units (CFUs) of *C. albicans* 48 h after being treated with PDT using FLG-Ce6 as the photosensitizer is highly reduced, as shown in Figure 4b. The photoactivation of *C. albicans* without a photosensitizer does not cause any change in the cell viability, nor does the incubation with any of our photosensitizers without light exposure. However, when *C. albicans* is exposed to Ce6 and then photoactivated by 15 min of irradiation with the LEDs, their viability is reduced up to 10%. Astonishingly, when *C. albicans* is exposed to FLG-Ce6 and is photoactivated for 15 min, its viability is reduced by more than 95%. This reduction in the viability was not observed when the cells were incubated with the FLG-Ce6 hybrid nanomaterial without photoactivation. This confirms that the viability effects are due to the ROS generation by the photosensitizer in each case, where FLG-Ce6 hybrid nanomaterial has a better photosensitizer effect in PDT against *C. albicans*, as compared Ce6 alone, which proves that graphene enhances the photosensitizing properties of Ce6. This may be due to the electron/energy donor effect of graphene sheets on the Ce6 molecules, facilitating their photoexcitation and increasing the amount of ROS generated. Another important factor contributing to the increased ROS generation is the modified spatial arrangement of the Ce6 molecules, which tends to evenly distribute the molecules over the surface of FLG. This is in contrast to the high interaction in the water solution. And finally, another contributor to the increased ROS generation is the ability of FLG to absorb red and NIR light. This modified spatial arrangement effectively increases the volume of the sample accessible to the system for the interaction with molecular oxygen. Also, FLG can delay the photobleaching of Ce6 when forming a hybrid and exposed to red light. The protection of Ce6 by FLG from photobleaching could occur by various mechanisms. One of such photobleaching mechanism occurs when the photosensitizer reacts with the generated ROS, creating oxygenated adducts on the structure of the molecules. In this case, the graphene sheets protect the Ce6 molecules by shielding half of the photosensitizer surface area, due to their π–π interactions. Besides this, the high surface area of the graphene sheets can act as a hiding place for the Ce6 molecules – having more surface area available, the graphene sheets can react more readily with the ROS that accumulate in its surroundings.

## Conclusion

The FLG-Ce6 hybrid nanomaterial presented in this work was shown to enhance the capacity for the generation of ROS species compared with Ce6 alone, as corroborated by measuring the fluorescence intensity produced by the reporter SOSG. This hybrid nanomaterial does not present quenching of the fluorescence during the 15 min irradiation time, suggesting that FLG protects Ce6 from photobleaching. We demonstrated that the viability of *C. albicans* is drastically reduced when treated with the FLG-Ce6 hybrid nanomaterial and exposed to red light (632 nm) for 15 min. The viability was measured 48 h after photoactivation, and we found an antifungal effect only when exposed to the light source. This shows that *C. albicans* is rendered almost completely unviable after exposure to ROS and not after its incubation with the FLG-Ce6 hybrid nanomaterial without light exposure. An increased PDT effect was observed with the FLG-Ce6 hybrid nanomaterial by lowering the viability of *C. albicans* up to 95%. In contrast, Ce6 alone lowers the viability of *C. albicans* to only 10% under the same irradiation conditions.

These results are very promising for the development of new ways of applying PDT. More studies must be done in order to determine the toxicity of this new generation of nanostructured photosensitizers, such as the hybrid nanomaterial presented in this work. However, immediate applications of the hybrid nanomaterial, for example, the coating of medical instruments or medical-grade supplies (such as sheets, tubes, robes) to facilitate their sterilization, thus maintaining a clean and fungi-free environment and preventing the occurrence of nosocomial infections, are farsighted.

## Experimental

### Synthesis of a sterile graphene/chlorin e6 hybrid material

The few-layer graphene/chlorin e6 hybrid material (FLG-Ce6) was prepared using the method reported by Hernández et al. [36]. All solvents and chemicals were obtained from commercial suppliers and used without further purification. Chlorin e6 (Ce6) and graphite were purchased from Frontier Scientific Logan and Bay Carbon, Inc., respectively. 1 mg of graphite and 2 mL of a solution of Ce6/methanol at 1 mg/mL was added to 8 mL of deionized water. The sample was sonicated for 45 min using a Branson 2510 ultrasonic bath with a frequency of 40 kHz and power of 130 W, then centrifuged at 500 rpm for 90 min. The supernatant was filtered and washed with deionized water and resuspended in 10 mL of deionized water. The synthesis methodology was always carried out under sterile conditions.

### Characterization

The UV–vis spectroscopy characterization was carried out with a Cary 60 UV–visible spectrophotometer using 10 mm long quartz cuvettes. The Raman spectra were obtained by means of a Thermo Scientific DXR Raman Microscope equipped with a diode-pumped solid-state laser (DPSS) at wavelength of 532 nm as the excitation source. A 10× objective with a 50 µm slit aperture and 5 s of exposure time were used. The laser power impinging on the sample was between 5 and 10 mW, the spatial resolution was 2 cm^−1^ and the spot size was ≈1 µm^2^. The samples were recorded from drops of the dispersions deposited on clean silicon wafers and left to dry under vacuum. The chemical composition of the samples was investigated using X-ray photoelectron spectroscopy (XPS) with a VERSAPROBE PHI 5000 instrument from Physical Electronics, equipped with a monochromatic Al Kα X-ray source under ultrahigh vacuum conditions. The energy resolution was 0.7 eV. For the compensation of built-up charge on the sample surface during the measurements, dual beam charge neutralization composed of an electron gun (≈1 eV) and an argon ion gun (≤10 eV) was used. The XPS spectra were deconvoluted using commercially available software (CASA-XPS). TEM images were obtained using a JEOL JEM-2100 instrument with a voltage acceleration of 200 kV. The preparation of the samples was done by the “drop casting” technique by depositing 100 μL of the FLG-Ce6 solution on the TEM grids (200 mesh, cooper, carbon only) and drying them in vacuum for 48 h.

### Photoactivation

The illumination source consists of 6 LEDs connected in parallel and distributed as follows: five were placed around a cylindrical container and one at the bottom of the container as shown in Figure 4c. The device is powered by a voltage source of 2.3 V and the emission spectrum of the LED light is centered at 632 nm with a full width at half maximum (FWHM) of 16 nm. The power measured just at the output of each LED is approximately 80 mW, however the light diverges and part of this light is lost before reaching the sample. On average, the light source provides a total power in the center of the sample of approximately 150 mW.

### Singlet oxygen production assays

We carried out a test in the presence 1 µM of a reporter molecule called singlet oxygen sensor green reagent (SOSG) to corroborate that FLG-Ce6 has the capacity to produce singlet oxygen and to quantify the production of the radical. The samples of FLG-Ce6 and Ce6, with the same concentration of Ce6, were subjected to a photoactivation test, by exposing them to the LED array source. The samples were illuminated for 5, 10 and 15 min, and the fluorescence of the SOSG reporter was measured in each case.

### Photosensitizer effect of FLG-Ce6 and Ce6 in PDT against *C. albicans*

We used the strain ATCC 90028 of *C. albicans* to evaluate the effect of FLG-Ce6 as a photosensitizer in PDT. FLG-Ce6 with 1.5 µg of Ce6 (this is the concentration of Ce6 in the hybrid and the total amount in the sample) was added to 2 mL of PBS containing 1 × 10^5^ cells of *C. albicans*. The samples were shaken for 3 h at 37 °C. Afterwards, the samples were centrifuged for 5 min at 1500 rpm and the supernatant was discarded. The pellet was resuspended in 2 mL of PBS. Following this, the samples were exposed to light for 15 min by the LED array. 80 μL of each sample was seeded in dextrose Sabouraud agar plates. The dishes were incubated for 48 h at 37 °C. The evaluation of the viability of *C. albicans* was measured by counting colony-forming units (CFUs) on the plates. The same procedure was done for the samples where the Ce6 molecule alone was used as the Ps.

## References

[1] Pfaller M A, Diekema D J, Turnidge J D, Castanheira M, Jones R N (2019). Open Forum Infect Dis.

[2] Donnelly R F, McCarron P A, Tunney M M (2008). Microbiol Res.

[3] Eggimann P, Garbino J, Pittet D (2003). Lancet Infect Dis.

[4] Acosta S, Quintana M (2019). Graphene for Photodynamic Therapy. Nanoscale Materials for Warfare Agent Detection: Nanoscience for Security.

[5] Issa Z, Hamblin M R, Rai M, Kon K (2015). Photodynamic Therapy of Infectious Disease Mediated by Functionalized Fullerenes. Nanotechnology in Diagnosis, Treatment and Prophylaxis of Infectious Diseases.

[6] Brown S B (2003). J Dermatol Treat.

[7] Bashkatov A N, Genina E A, Kochubey V I, Tuchin V V (2005). J Phys D: Appl Phys.

[8] Kalka K, Merk H, Mukhtar H (2000). J Am Acad Dermatol.

[9] Dong J, Toh H J, Thong P S P, Tee C S, Bi R, Soo K-C, Lee K (2014). J Photochem Photobiol, B.

[10] Jeon Y-M, Lee H-S, Jeong D, Oh H-K, Ra K-H, Lee M-Y (2015). Life Sci.

[11] Wang H, Wang X, Wang P, Zhang K, Yang S, Liu Q (2013). Ultrasound Med Biol.

[12] Huang L, Zhiyentayev T, Xuan Y, Azhibek D, Kharkwal G B, Hamblin M R (2011). Lasers Surg Med.

[13] Xue Q, Wang X, Wang P, Zhang K, Liu Q (2015). Photodiagn Photodyn Ther.

[14] Yang X, Wang D, Shi Y, Zou J, Zhao Q, Zhang Q, Huang W, Shao J, Xie X, Dong X (2018). ACS Appl Mater Interfaces.

[15] Huang P, Xu C, Lin J, Wang C, Wang X, Zhang C, Zhou X, Guo S, Cui D (2011). Theranostics.

[16] Amirshaghaghi A, Yan L, Miller J, Daniel Y, Stein J M, Busch T M, Cheng Z, Tsourkas A (2019). Sci Rep.

[17] Tian B, Wang C, Zhang S, Feng L, Liu Z (2011). ACS Nano.

[18] Yang Y, Asiri A M, Tang Z, Du D, Lin Y (2013). Mater Today.

[19] Mata-Cruz I, Vargas-Caamal A, Yañez-Soto B, López-Valdivieso A, Merino G, Quintana M (2017). Carbon.

[20] Schedin F, Lidorikis E, Lombardo A, Kravets V G, Geim A K, Grigorenko A N, Novoselov K S, Ferrari A C (2010). ACS Nano.

[21] Hernández-Sánchez D, Villabona-Leal G, Saucedo-Orozco I, Bracamonte V, Pérez E, Bittencourt C, Quintana M (2018). Phys Chem Chem Phys.

[22] Jaleel J A, Sruthi S, Pramod K (2017). J Controlled Release.

[23] Zhou L, Jiang H, Wei S, Ge X, Zhou J, Shen J (2012). Carbon.

[24] Fan B, Guo H, Shi J, Shi C, Jia Y, Wang H, Chen D, Yang Y, Lu H, Xu H (2016). J Nanosci Nanotechnol.

[25] Akbari T, Pourhajibagher M, Hosseini F, Chiniforush N, Gholibegloo E, Khoobi M, Shahabi S, Bahador A (2017). Photodiagn Photodyn Ther.

[26] Gurunathan S, Han J W, Eppakayala V, Kim J-H (2013). Colloids Surf, B.

[27] Qu G, Wang X, Liu Q, Liu R, Yin N, Ma J, Chen L, He J, Liu S, Jiang G (2013). J Environ Sci.

[28] Wilczek P, Major R, Lipinska L, Lackner J, Mzyk A (2015). Mater Sci Eng, C.

[29] Pinto A M, Gonçalves C, Sousa D M, Ferreira A R, Moreira J A, Gonçalves I C, Magalhães F D (2016). Carbon.

[30] Lopes M, Alves C T, Raju B R, Gonçalves M S T, Coutinho P J G, Henriques M, Belo I (2014). J Photochem Photobiol, B.

[31] Machado-de-Sena R M, Corrêa L, Kato I T, Prates R A, Senna A M, Santos C C, Picanço D A, Ribeiro M S (2014). Photodiagn Photodyn Ther.

[32] Azizi A, Amirzadeh Z, Rezai M, Lawaf S, Rahimi A (2016). J Photochem Photobiol, B.

[33] Hernandez Y, Nicolosi V, Lotya M, Blighe F M, Sun Z, De S, McGovern I T, Holland B, Byrne M, Gun'Ko Y K (2008). Nat Nanotechnol.

[34] Lotya M, Hernandez Y, King P J, Smith R J, Nicolosi V, Karlsson L S, Blighe F M, De S, Wang Z, McGovern I T (2009). J Am Chem Soc.

[35] Quintana M, Tapia J I, Prato M (2014). Beilstein J Nanotechnol.

[36] Hernández-Sánchez D, Scardamaglia M, Saucedo-Anaya S, Bittencourt C, Quintana M (2016). RSC Adv.

